# Optimization of saline wastewater treatment using electrochemical oxidation process: Prediction by RSM method

**DOI:** 10.1016/j.mex.2019.03.015

**Published:** 2019-04-16

**Authors:** Mohammad Darvishmotevalli, Ahmad Zarei, Maryam Moradnia, Mohammad Noorisepehr, Hamed Mohammadi

**Affiliations:** a Department of Environmental Health Engineering, School of Health, Isfahan University of Medical Sciences, Isfahan, Iran; b Department of Environmental Health Engineering, Faculty of Health, Social Development and Health Promotion Research Center, Gonabad University of Medical Sciences, Gonabad, Iran; c Department of Health Research Center, Kurdistan University of Medical Sciences, Sanandaj, Iran; d Department of Environmental Health Engineering, Public Health School, Alborz University of Medical Sciences, Alborz, Iran; e Research Center for Health, Safety and Environment (HSE), Alborz University of Medical Sciences, Alborz, Iran; f Assistant Professor, Torbat Jam Faculty of Medical Sciences, Torbat jam, Iran

**Keywords:** Optimization of saline wastewater treatment using electrochemical oxidation process: Prediction by RSM method, Saline wastewater, Electrochemical oxidation, RSM, Optimization

## Abstract

Response surface methodology (RSM) was applied to find the optimum parameters for COD and TOC removal from saline wastewaters using electrochemical oxidation process. The independent variables considered were reaction time, pH, salt concentration, and voltage. Optimization of parameters was performed by analysis of variance (ANOVA). Quadratic regression equation was suggested as a model for prediction of chemical oxygen demand (COD) and total organic carbon (TOC) removal efficiency. The results indicated that the COD and TOC removal efficiencies at the optimal conditions of pH 7.69, reaction time of 30.71 min, salt content of 30. 94 g/L and voltage of 7.41 V were 91.78% and 68.49%, respectively. In terms of COD and TOC removal efficiency, the coefficients of determination were found to be 0.95 and 0.94, respectively. This study suggests that electro-oxidation is an effective process in decreasing COD and TOC from saline wastewaters. Further, RSM was a suitable technique for optimization of the variables involved in COD and TOC removal through electro-oxidation process.

• The findings demonstrate that response surface methodology is a good tool for the optimization of parameters of the experimental data.
• A quadratic model was suggested as a good model for COD and TOC removal prediction.
• The findings proved good agreement between the experimental data and the predicted equation.

## Graphical abstract

**Figure:**
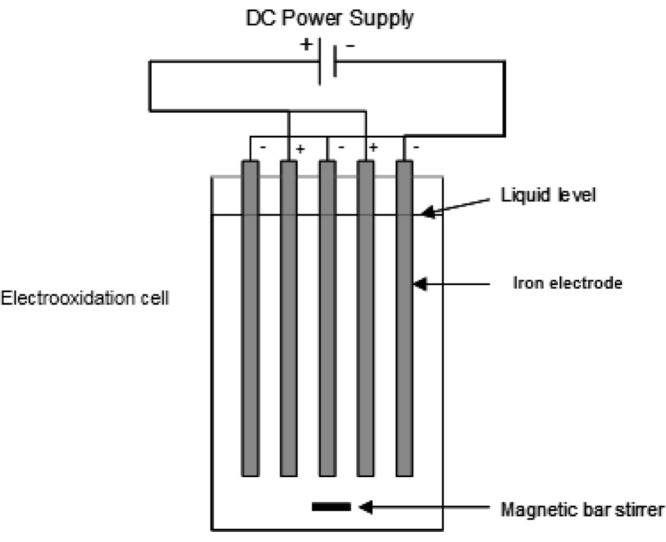

**Specifications Table**

**Table:** 

Subject Area:	Environmental Sciences
More specific subject area:	Electrochemical oxidation process
Method name:	Optimization of saline wastewater treatment using electrochemical oxidation process: Prediction by RSM method
Name and reference of original method:	O. Lefebvre, R. Moletta. Treatment of organic pollution in industrial saline wastewater: a literature review. Water Res. 40 (2006) 3671–3682.
Resource availability:	The data are available with this article

## Method details

Wastewater generation has witnessed an ascending trend parallel the development of industrialization, urbanization and rapid growth of population around the world [[1], [2], [3], [4], [5]]. Several types of industrial wastewaters including pharmaceuticals, tannery and leather, textile, meat processing, fish, and marine products contain high concentrations of salts [6,7]. Salt can also be found at high concentrations in the leachate of urban landfill sites, contaminated groundwater, and the wastewaters resulting from mining operations and recycling units of gas and oil industries [[8], [9], [10]]. Fish processing industries need large amounts of sodium chloride for fish preservation. The wastewater produced by such industries contains large amounts of nitrogenized organic compounds and salts [[11], [12], [13], [14]]. Leather industries in tanning process require high concentrations of salt for removing hair and wool from the animal skins. Moreover, the concentration of salt in the effluent of extraction, refinery, and oil processing industries is very variable, such that in some cases it is several times higher than the concentration of salt in seawater [15].

Application of biological treatment processes for the saline wastewaters has always faced many problems. In wastewaters containing high salt (higher than 1 wt%), due to the dehydration of microbial cells, it is virtually impossible to use microbial treatment. Therefore, salt removal will be the prerequisite of biological treatment in the saline wastewaters [[16], [17], [18], [19], [20]]. However, the salt removal from wastewater in conventional systems is rarely possible. Membrane treatment techniques including ultrafiltration [21], nanofiltration [22], reverse osmosis [23], and advanced oxidation processes [24] including electro-Fenton and electrolysis have been studied for the treatment of wastewaters with high salt content. Due to the high content of cations and anions, these wastewaters have a high electrical conductivity. Therefore, electron-assisted processes can be suitable options for the treating such wastewaters. Electro-oxidation process have been successfully used for the treatment this group of effluents such as the textile and tanning wastewaters along with the domestic wastewater and also the leachate of landfills [25,26].

In classic optimization method, one variables changes at a time, while other parameters are kept constant [[27], [28], [29]]. But the classic method is not able to determine the complex interaction between the variables and responses [30,31]. RSM has been derived from statistical and mathematical techniques which can be used for studying the effect of different factors at various levels and their interactions [32]. This method consists of four main stages including experiment design, model fitting, model verification, and determining the optimal conditions. The central composite design (CCD) is one of the most frequently used technique among RSM due to the need for fewer number of experiments [[33], [34], [35]]. The aim of this study was to optimize the variables which affect the electrochemical oxidation process to improve the treatment of saline wastewater using RSM.

## Materials and methods

### Experimental setup and procedure

**Fig. 1:**
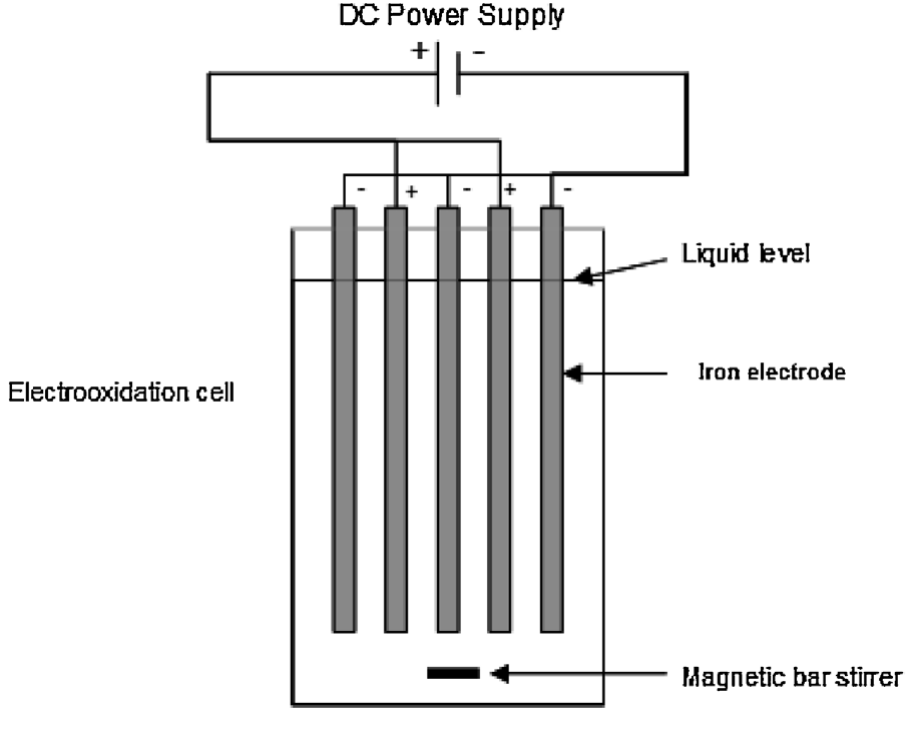
Schematic of the electro-oxidation reactor used in this study.

This research is an experimental study which was performed in a rectangular batch reactor made of plexiglas. The net volume of this reactor was 1 L with dimensions of 15 × 6 × 16 cm. Iron columns with a diameter of 1.6 cm and length of 14 cm were connected to each other by some pieces of cable and then placed inside the reactor as two series of parallel electrodes (Fig. 1). Each of the aluminum electrodes series was connected to direct electric current. The electric current was provided by an AC/DC current exchanger. In order to prepare synthetic wastewater with a given salt content and sodium chloride was used. The resulting concentration of COD and TOC were 3500 and 2000 mg/L, respectively. To adjust the pH, 1 N NaOH and HCl solutions (Merck Co.) were used. All chemical experiments were performed according to the standard instructions [36]. The performance of the process was evaluated based on the responses of COD and TOC removal efficiencies [[37], [38], [39]] (Eq. (1)).

(1) $$\mathrm{COD}\;\mathrm{or}\;\mathrm{TOC}\;\mathrm{removal}\;\mathrm{efficiency}\;\left(\%\right)=\;\;\left(\frac{c_{i}-c}{c_{i}}\right)\;\times 100$$

That C_i_ and C are initial and final concentrations of COD or TOC (mg/L), respectively.

RSM was used to optimize variables influencing the removal of COD and TOC from saline solutions. Three independent variables including pH, reaction time, salt concentration and voltage at three coded levels (−1, 0, +1) were examined (Table 1). The ranges of variables were obtained from preliminary screening experiments and literature reviews.

**Table 1:** Experimental levels of independent process variables.

Independent variables	Coded levels		
	−1	0	+1
pH	4	6	8
Reaction time (min)	20	30	40
Salt concentration (g/L)	12	22	32
Voltage (V)	3	6	9

In this research, the experimental design was based on two-level full factorial design to which central and star points were also added. The total number of experiments (N) can be calculated according to Eq. (2).

(2) N = N_a_ + N_0_ + N_c_

Where, N_a_ represents the number of two-level experiments in a full factorial design (2^4^), N_0_ is the number of replication in the central point (5 replications) for evaluation of net error, and N_c_ denotes the number of star points (2 × 4). Therefore, in total 29 experiments were designed. Statistical design of the experiments and data analysis was performed using the Design Expert 7 software. To determine the optimal values of the independent variables of the process, two dependent variables of COD and TOC removal were analyzed as the response. Second-order model equation for prediction of the optimal conditions can be expressed by the following equation:

(3) $$\;Y=\beta_{0}+\sum_{i=1}^{K}\beta i∙Xi+\sum_{i=1}^{K}\beta ii∙{Xi}^{2}+\sum_{ii\leq j}^{K}\sum_{j}^{K}\beta ij.Xi.Xj+\ldots +e$$

Where Y, is the response variable, i, j, and β are the linear, second-order, and regression constant, respectively, e is random error and k is the number of parameters. All the variables were optimized in the experiments. For analysis of the data and determining the interactive effects between the independent variables of the process and responses, ANOVA was performed. To prevent systemic error, the experiments were performed randomly. The coefficients of the second-order model, which interpret the amount of removal of the studied parameters (responses) act as the performance of independent variables (factors). The research data were analyzed by multiple regressions. The coefficients were analyzed using analysis of variance and p ≤ 0.05 was determined as the significance level.

The quality of the model fitting was controlled by determination coefficients (R^2^ and Adj.R^2^), while the statistical significance was controlled by Fischer test (*F*-test) [40]. The desired objectives were set as maximum removal of COD and TOC.

## Results

The results of experiments in the form of removal rate of COD (Y_1_) and TOC (Y_2_) are provided in Table 2. The removal efficiency of COD and TOC varied within the ranges of 36–89% and 30–67%, respectively.

**Table 2:** Face central composite design (FCCD) for COD and TOC removal.

Run	pH	Time (min)	Salinity (gr/L)	Voltage (volt)	COD removal (%)	TOC removal (%)
					Actual	Actual
1	6	30	22	6	80	67
2	6	30	32	9	81	62
3	6	30	12	3	43	39
4	8	20	22	6	65.2	43.1
5	4	30	22	3	38	36
6	6	40	12	6	52	37
7	6	20	32	6	57	32
8	8	40	22	6	76	44
9	6	30	22	6	83	62
10	6	30	22	6	81	67
11	6	40	32	6	73	54
12	6	30	32	3	51	37
13	8	30	22	3	71	52
14	8	30	22	9	81.5	58.2
15	6	30	22	6	79.4	61.7
16	4	30	22	9	69	55
17	8	30	12	6	57.5	42
18	6	40	22	9	83	60
19	6	30	22	6	86	66
20	6	20	22	9	43	40
21	6	30	12	9	52	40
22	4	40	22	6	60	37
23	8	30	32	6	89	64.5
24	6	20	12	6	36	30
25	6	40	22	3	53	31
26	4	20	22	6	49	37
27	4	30	32	9	51	40
28	6	20	22	3	51	30
29	4	30	12	6	49	37

The results obtained from ANOVA for COD and TOC removal efficiency responses are given in

The results of ANOVA suggest that the both obtained second-order models are significant, as the probability values in them are low (p ≤ 0.0001). According to the results, 12 out of the 14 model terms were significant for COD and TOC removal efficiency which include: pH (A), reaction time (B), salt concentration (C), voltage (D), square terms of pH (A^2^), reaction time (B^2^), salt concentration (C^2^), and voltage (D^2^), and interaction terms of AC, BC, AD, BD and CD.

After elimination of the terms which were not statistically significant, the modified quadratic model, for COD and TOC removal efficiency were obtained as Eqs. (5) and (6), respectively. The terms in the models are in a coded format.

(4) *Y1 = 81.88 + 7.52A + 6.98B + 11.96C + 10.13D + 4.38AC + 6.75BD + 6.75BC + 5.25CD −* *10.63A^2^* − .63B*^2^* *−* *8.59C^2^* *−* *13.97D^2^*

(5) *Y2 = 64.74 + 5A + 4.24B + 5.38C + 7.52D + 4.88AC+3.75BC + 4.75BD + 6CD* − *7.39A^2^* − *16.22B^2^* − *11.3C^2^* − *8.09D^2^*

## Discussion

The coefficient of determination (R^2^) which represents the ratio of the total changes in the predicted response by the model shows the sum of squares regression (SSR) to the total sum of squares (SST) ratio. Largeness of R^2^ and its closeness to 1 is desirable and a desired correspondence with adjusted R^2^ (Adj.R^2^) is necessary. The quality of fitness of second-order polynomial model is expressed by R^2^ [37,41,42]. In this study, R^2^ for the removal of COD and TOC was 0.97 and 0.94, respectively, while Adj.R^2^ was 0.95 and 0.89, respectively. All of the values of R^2^ were above 0.8. According to Mirhosseini et al. [43], for a good fitness of model, R^2^ should be at least 0.8. Bashir et al. [44] reported that high R^2^ values suggest a great accordance between the experimental data and data estimated by the model. Therefore, high R^2^ values and their accordance with Adj.R^2^ in this study suggest the high significance of the model.

The Adequate precision (AP) that has been shown in Table 3 is “signal-to-noise ratio” index. In other word, AP compares the range of values predicted at design points with the mean prediction error. Ratios above 4 suggest precision of the signal for models to find design space [41], which in this study for COD and TOC removal were 23.21 and 12.38%, respectively that implies the existence of sufficient signal and the high power of the model in prediction of the results. Lack of fit test describes the changes in data around the fitted model. If the model has not been fitted well, this test is significant [45]. The values of lack of fit test related to the second-order model fitted for COD and TOC removal responses were 0.3172 and 0.1749, confirming the data fitness on the model.

**Table 3:** ANOVA for COD and TOC removal efficiency using electro-oxidation process.

Source	Sum of squares		df		Mean squares		F- value		P-value	
	COD Removal (%)	TOC Removal (%)	COD Removal (%)	TOC Removal (%)	COD Removal (%)	TOC Removal (%)	COD Removal (%)	TOC Removal (%)	COD Removal (%)	TOC Removal (%)
Model	6788.24	4246.27	12	12	565.69	353.86	29.66	21.81	<0.0001	<0.0001
A-pH	*678*	318.27	1	1	678	318.27	35.55	19.61	<0.0001	0.0004
B-time	585.2	215.9	1	1	585.2	215.9	30.69	13.30	<0.0001	0.0022
C-salinity	1716.02	346..69	1	1	1716.02	346.69	89.98	21.36	<0.0001	0.0003
D-voltage	1230.19	678	1	1	1230.19	678	64.51	41.78	<0.0001	<0.0001
AC	76.56	95.06	1	1	76.56	95.06	4.01	5.86	0.0064	0.0278
BC	182.25	56.25	1	1	182.25	56.25	9.56	3.47	0.007	0.00811
BD	182.25	90.25	1	1	182.25	90.25	9.56	5.56	0.007	0.0314
CD	110.25	144	1	1	110.25	144	5.78	8.87	0.0278	0.0089
A^2^	733.18	353.92	1	1	733.18	353.92	38.45	21.81	<0.0001	0.0003
B^2^	601.74	1707.4	1	1	601.74	1707.4	31.55	105.22	<0.0001	<0.0001
C^2^	479.09	828.14	1	1	479.09	828.14	25.12	51.03	<0.0001	<0.0001
D^2^	1265.76	424.18	1	1	1265.76	424.18	66.37	26.14	<0.0001	0.0001
Residual	305.13	259.64	16	16	19.07	16.23				
Lack of fit	276.44	231.09	12	12	23.04	19.26	3.21	2.70	0.1351*	0.1749*
Pure error	28.69	28.55	4	4	7.17	7.14				
Cor. total	7093.37	4505.91	28	28						

Other statistical parameters						
	R^2^	Adj. R^2^	A.P	S.D	C.V	PRESS
COD removal	0.95	0.92	16.65	4.37	6.81	1217.64
TOC removal	0.94	0.89	12.38	4.03	8.58	907.58

**Fig. 2:**
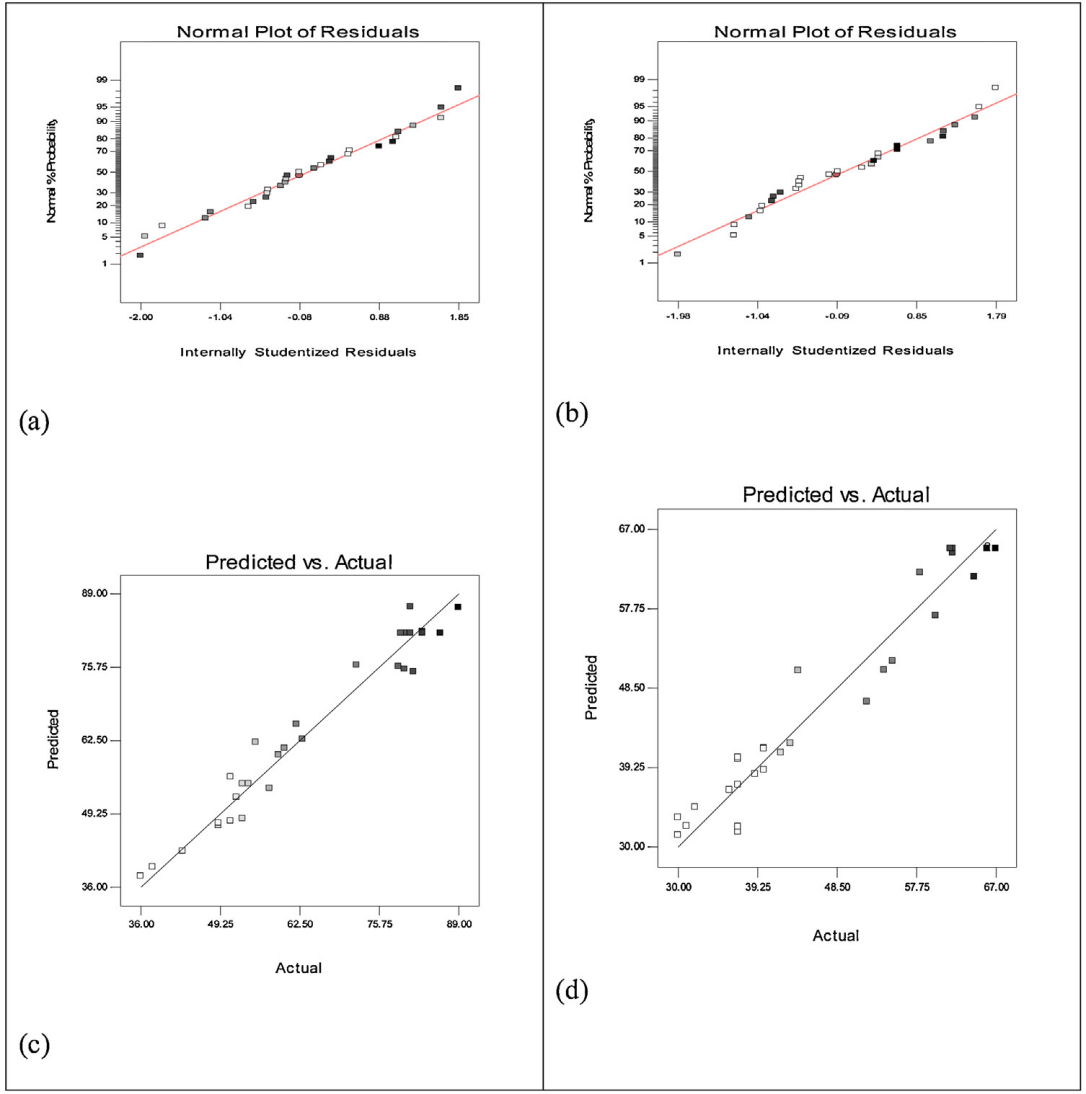
Normal probability plots of the studentized residuals for COD and TOC removal efficiency: (a, b), and residuals versus run plots for COD and TOC removal efficiency (c, d).

Furthermore, the model's adequacy can be evaluated using diagnostic diagrams including normal probability distribution diagram of residuals, the diagram of predicted values versus real values. Fig. 2 shows the distribution of normal probability percentage versus studentized residuals for COD (Fig. 2a) and TOC (Fig. 2b) removal levels. Further, Fig. 2c indicates the distribution of residuals versus fitted values for COD (Fig. 2c) and TOC (Fig. 2d) removal levels. As can be seen in these diagrams, the points lie on a relatively straight line, suggesting the constancy of the variance and normal distribution. In the normal probability distribution diagram of residuals, the points are aligned along an almost straight line. Some of the scattered points are even expected in normal distribution of the data. According to Fig. 2, good correlations between predicted values and real values regarding COD and TOC removal confirm the adequacy of the models in predicting the removal of these two pollutants.

The interactive reaction between four independent variables and dependent variables (responses) can be plotted based on regression models (Eqs. (3) and (4)) and aligned diagrams of the interactive relationships between them and the response variable.

**Fig. 3:**
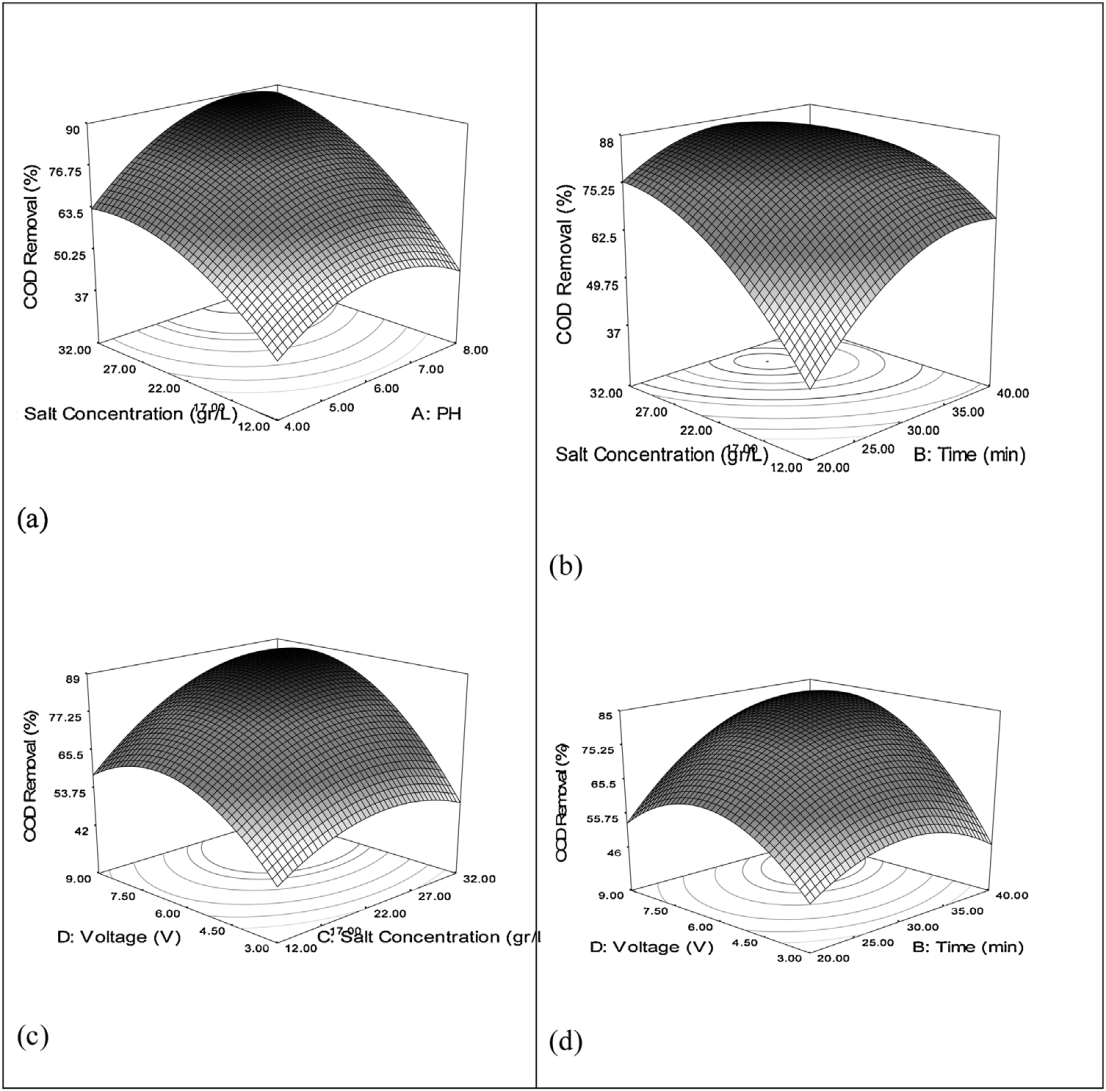
Relation between COD removal efficiency and the interaction terms by 3D plot: (a) interaction between pH- salt concentration (b), interaction between salt concentration-time (c), interaction between voltage- salt concentration, and (d) interaction between voltage- time.

**Fig. 4:**
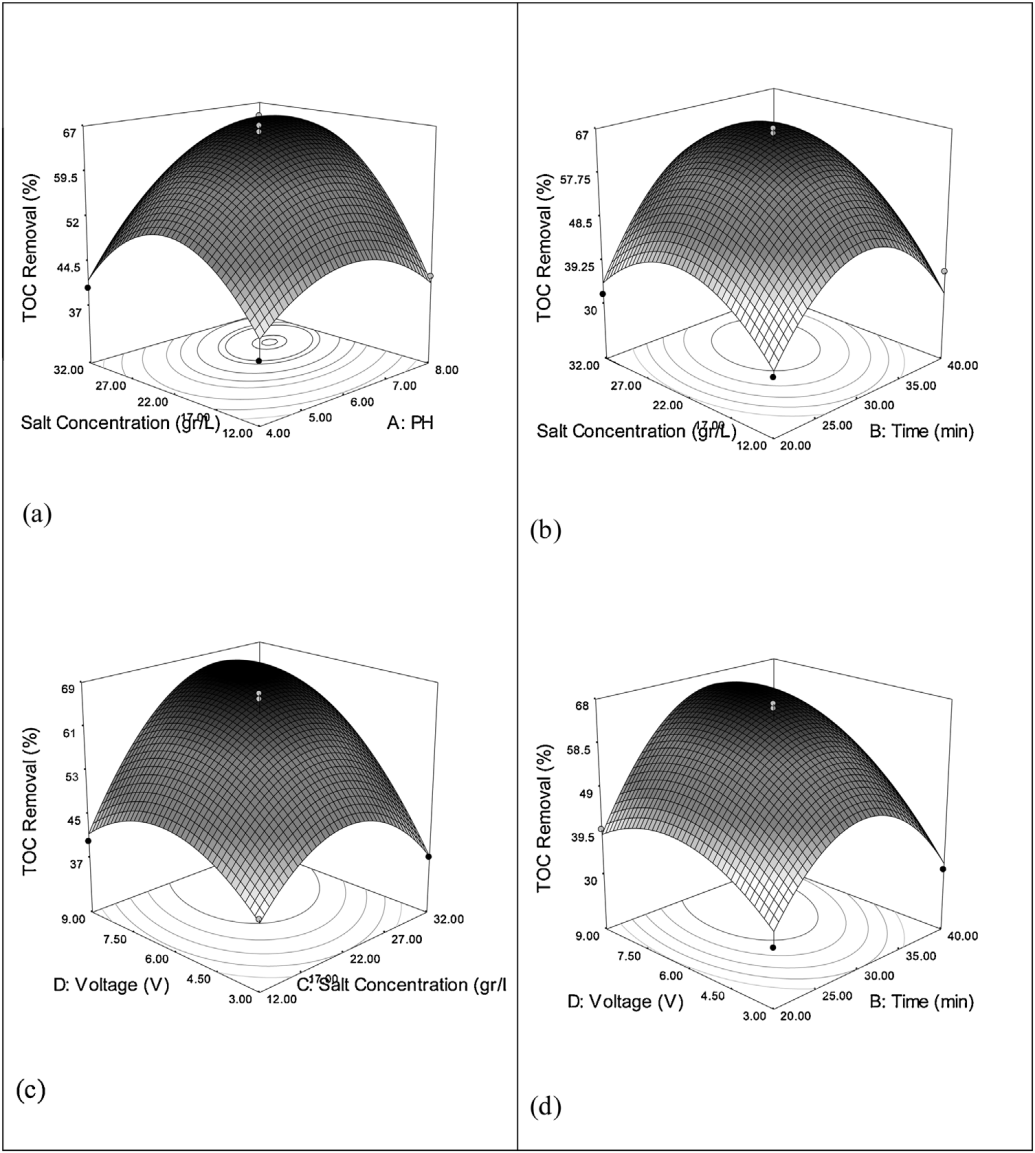
Relation between TOC removal efficiency and the interaction terms by 3D plot: (a) interaction between pH-salt concentration (b), interaction between salt concentration-time (c), the interaction between voltage- salt concentration, and (d) the interaction between voltage-time.

Fig. 3, Fig. 4 indicate the interactive effects between the variables influencing COD and TOC removal.

Figs. 3a and 4 a demonstrate the effect of changes in pH and salt concentration on the reduction of COD and TOC. In these conditions, the voltage and reaction time have been kept at optimal values. As can be seen in these figures, with the increase in the salt concentration, COD and TOC removal percentage increases, which is due to indirect oxidation caused by production of chloride or hypochlorite in response to electrolysis process of NaCl occurring in the reactor, which together with produced hydroxyl radical, causes degradation of organic compounds [46]. The optimum pH for the removal of COD and TOC were about 7 and 6.5 respectively. Also, the optimum salt concentration for the removal of COD and TOC were obtained at 29 and 24 g/L, respectively. In this condition, removal efficiencies for COD and TOC were 89% and 67%, respectively.

By increasing the pH values, COD and TOC removal percentage increases. However, this ascending trend is predicted for pH values up to 7, after which the removal percentage continues in a descending trend. During electrochemical treatment, chloride compounds including hypochlorite, chloride ion, chlorine gas, and chlorate are developed. In this research, the suitable pH was alkaline. In this alkaline environment, the chlorine gas produced from Cl^−^ ion at anode level and in the solution environment it is converted to hydrolyzed hypochlorite, according to the following reactions:

(6) 2Cl^−^ ↔ Cl_2_ + 2e^−^

(7) Cl_2_ + H_2_O ↔ HOCl + H^+^ + Cl^−^

(8) HOCl ↔ H^+^ + OCl^−^

Then, organic compound, according to reaction 4, is indirectly oxidized by hypochlorite ion.

(9) R + OCl^−^ ↔ CO_2_ + H_2_O + Cl^−^

In addition, under alkaline conditions, it mostly changes into hypochlorite, preventing formation of chlorine-based toxic compounds [47].

The results of a research by Kumar et al. regarding the treatment of nitrophenol using electro-oxidation process indicated that pH plays an important role in degradation of organic compounds. They also reported that with the increase in pH from 3.5 to 5.5, due to elevation of OH° radical in the medium of the reaction, COD and TOC removal percentage increases, and at optimal pH of 5.5, the removal percentage of COD and TOC was 96 and 82%, respectively. This efficiency remained constant up to pH 7 [48]. Considering the effect of pH on degradation of aromatic organic compounds by electrolytic processes, various reports have been published. Some researchers have reported that both alkaline and acidic conditions are suitable for degradation of aromatic organic compounds, whereas some others have stated that neutral conditions are suitable for degradation of organic compounds [49,50].

As can be seen in (Figs. 3b and 4 b), with the increase in the reaction time and salt concentration, COD and TOC removal efficiency finds an ascending trend. Considering the reaction time, the trend of changes follows the descending pattern after 30 min. The maximum removal percentage is observed, after which a descending trend in COD occurs. As can be seen in this figure, the changes in the COD removal efficiency within the range of changes in the two independent variables have cocentric points and one peak, which suggest that optimal points have been obtained. As can be seen in Figs. 3b and 4 b, the optimum reaction time and salt concentration for the removal of COD and TOC was observed at 30 min and 29 and 24 g/L, respectively. The removal efficiency of COD and TOC was 87% and 66%, respectively. After that, the removal trend decreases, because high concentration of salt in the reactor lead to production of chloride gas which leave the rector. Paniza and Krizola reported that chloride ion is an important parameter in evaluation of wastewater quality. Their results indicated that addition of 5 mg/L of chloride ion has a significant effect in reducing COD of this type of wastewater [47]. The results of the study performed on oxidation of ammonia in wastewater by electro oxidation process, the optimal retention time for COD reduction was reported to be 30 min [51], which is in agreement with the results of the present research. According to the study, longer reaction times have a lower removal percentage, which might be due to sequestration of metal hydroxides at electrode level [52].

According to the (Figs. 3c and 4 c), the higher removal rates occurred in both high salt concentration and high voltage. The optimum was obtained at voltage 6 and salt concentration 27 g/L. At optimum conditions, the removal rates of COD and TOC were 89% and 68%, respectively. However, after this point, a descending trend was seen in removal efficiency.

Furthermore, (Figs. 3d and 4 d) indicate the interactive reaction between independent variables of voltage and reaction time. As can be observed in these figures, with the increase in voltage and reaction time, COD and TOC removal efficiency increases and up to voltage of 6 V. This can be attributed to the effects of high voltage and sequestration of metal hydroxides on the surface of electrodes [52].

Xue et al. used electro-oxidation process for degradation of Perfluorooctanoic acid at a concentration of 100 micro m/L. In this research, a voltage of 0–4 V was used. The results showed that the maximum removal percentage was obtained at the voltage of 3.5–4 V. Further, the optimal voltage value was reported to be 7 [53]. This difference might be due to the low concentration of Perfluorooctanoic acid. Moreover, as voltages above 4 have not been studied, thus it cannot be judged reliably. The research by Zhio et al. has also reported 3.37 V for degradation of Perfluorooctanoic acid [54].

### Optimization of the operational conditions for the process and verification of experimental results

Optimization of COD and TOC removal for determining optimized points for operational conditions and achieving the maximum removal percentage was performed by estimation models 4 and 5. To achieve the highest removal performance at operational conditions of independent variables, COD and TOC removal percentage were selected at maximum value. The target values of four independent variables including reaction time, pH, salt concentration, and voltage were selected in in-range state. The values of optimal conditions for independent variables were obtained as follows: pH = 7.69, reaction time of 30.71 min, salt concentration of 30.94 g/L and voltage of 7.41 V. Under these conditions, the degree of desirability of the model was equal to 1, while the removal percentage of COD and TOC was 91.78% and 68.49%, respectively. To confirm the adequacy of the models and accuracy of the optimization method, 5 additional experiments were performed at the obtained optimal conditions (Table 4). The removal percentages obtained from the experiments and estimated by the models, for both response variables, have a close accordance with each other, confirming the accuracy of the approach in the models.

**Table 4:** Verification of experimental results at optimum conditions.

Optimum condition	COD removal efficiency (%)	TOC removal efficiency (%)
Experimental results	89.92%	67.12%
Model response	91.78%	68.49%
Error	1.86	1.37
Standard deviation	±1.37	±0.96

## Conclusions

The results of this study demonstrated that response surface methodology is a good tool for optimizing of parameters found from the experimental data. A quadratic model was suggested as a good model for the prediction of COD and TOC removal. Furthermore, ANOVA analysis indicated that pH (A), reaction time (B), salt concentration (C), voltage (D), square terms of pH (A^2^), reaction time (B^2^), salt concentration (C^2^), and voltage (D^2^), and interaction terms of AC, BC, AD, BD and CD had significant effects on COD and TOC removal efficiency. The optimum conditions were found at pH = 7.69, reaction time of 30.71 min, salt concentration of 30. 94 g/L and voltage of 7.41 V while at optimum conditions, the COD and TOC removal efficiency were found to be 91.78% and 68.49%, respectively. The findings proved a good agreement between the experimental data and the predicted equation. Therefore, the RSM can be proposed as a useful tool for the optimization of saline wastewater treatment using electrochemical oxidation processes.

## Conflict of interests

Authors have no conflict of interests.
